# The long noncoding RNA lnc-H19 is important for endurance exercise by maintaining slow muscle fiber types

**DOI:** 10.1016/j.jbc.2023.105281

**Published:** 2023-09-22

**Authors:** Yongqi Yue, Yanru Yue, Zeyu Fan, Yingying Meng, Chenglong Wen, Yalong An, Ying Yao, Xiao Li

**Affiliations:** 1 Key Laboratory of Animal Genetics, Breeding and Reproduction of Shaanxi Province, College of Animal Science and Technology, Northwest A&F University, Shaanxi, China; 2 Key Laboratory of Livestock Biology, Northwest A&F University, Shaanxi, China

**Keywords:** lnc-H19, mitochondria, endurance exercise, myofiber type

## Abstract

Skeletal muscle consists of different muscle fiber types whose heterogeneity is characterized by different metabolic patterns and expression of MyHC isomers. The transformation of muscle fiber types is regulated by a complex molecular network in which long noncoding (lnc) RNAs play an important role. In this study, we found that lnc-H19 is more enriched in slow muscle fibers. *In vitro*, interference of lnc-H19 by siRNA significantly promoted the expression of fast muscle fiber gene *MyHC IIB* and inhibited the expression of the slow muscle fiber gene *MyHC I*, thereby leading to a fast muscle fiber phenotype. In addition, interference of lnc-H19 significantly inhibited mRNA expression of the mitochondrial genes, such as *COX5A*, *COX-2*, *UQCRFSL*, *FABP3*, and *CD36*. Overexpression of lnc-H19 resulted in an opposite result. *In vivo*, knockdown of lnc-H19 by AAV-shRNA-H19 suppressed the mRNA expression of the slow muscle fiber gene *MyHC I* and the protein expression of slow-MyHC. Simultaneously, mitochondria were reduced in number, swollen, and vacuolated. The activities of succinate dehydrogenase, lactic dehydrogenase, and superoxide dismutase were significantly inhibited, and malondialdehyde content was significantly increased, indicating that deficiency of lnc-H19 leads to decreased oxidative metabolism and antioxidant capacity in muscle. Furthermore, inhibition of lnc-H19 decreased the weight-bearing swimming time and limb suspension time of mice. In conclusion, our results revealed the role of lnc-H19 in maintaining slow muscle fiber types and maintaining exercise endurance, which may help to further improve the regulatory network of lnc-H19 in muscle function.

Skeletal muscle is an important component of animal body as well as the main source of dietary protein (1). It is composed of different types of myofibers (2, 3), which are mainly distinguished by the expression of myosin heavy chain subtypes and the pattern of ATP production (4, 5). Compared with fast muscle fibers, slow muscle fibers are characterized with slow contraction, fatigue resistance, higher oxidative activity, and more mitochondrial contents (6, 7). Previous studies have found that endurance training generally induces phenotypic transformation from glycolic types to oxidative types (8). Transition from fast to slow muscle fibers can be achieved by stimulating the expression of factor tightly related with mitochondria biogenesis, peroxisome proliferator–activated receptor gamma coactivator-1α, in slow muscle fibers (9). In the process of muscle growth and development, muscle fiber types will change in response to changes in muscle function, the occurrence of diseases, and the process of aging and other physiological activities (10). The different distribution of muscle fiber types plays an important role in exercise ability.

As a superstar in lncRNA family, lnc-H19 plays a key role in development and function of skeletal muscle. For example, H19-encoded microRNAs, miR-675-5p and miR-675-3p, can execute the prodifferentiation function in myogenesis (11). Xu *et al*. (12) also demonstrated that H19 could promote the differentiation of bovine muscle satellite cells by suppressing *Sirt1/FoxO1*. These discoveries demonstrate that H19 plays an important role in skeletal muscle differentiation and myogenesis through multiple pathways. Moreover, overexpression of lnc-H19 has been reported to counteract lipopolysaccharide-induced mitochondrial damage (13). Downregulation of H19 expression mediated by DNA methylation plays a crucial role in cardiomyocyte metabolic disorders, mainly inducing cardiac respiratory dysfunction by promoting mitochondrial autophagy (14). These results indicate that there is an interaction between lnc-H19 and mitochondria, and the latter largely determines the transformation of muscle fiber types. Therefore, we speculate that lnc-H19 may enhance the exercise endurance of mice by altering the function of mitochondria.

In this study, we found that lnc-H19 expression promoted the transformation of fast to slow muscle fibers *in vitro*, and repressed lnc-H19 expression *in vivo* leads to loss of slow fibers, thus impairing endurance exercise.

## Results

### Lnc-H19 was differentially expressed in fast and slow muscles

**Figure 1:**
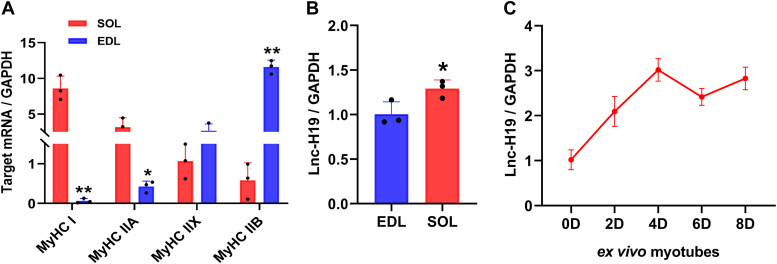
**Expression of lnc-H19 in different muscle and C2C12 cells during differentiation.***A*, expression of MyHC isoforms in SOL and EDL of mice (n = 3 biological replicates). *B*, Lnc-H19 expression levels in SOL and EDL of mice (n = 3 biological replicates). *C*, the expression of lnc-H19 during differentiation of C2C12 cells (n = 3 biological replicates with a minimum of two experimental replicates). The results are expressed as mean ± SD, n = 3, ∗*p* < 0.05 and ∗∗*p* < 0.01. EDL, extensor digitalis longus; lnc, long noncoding; SOL, soleus.

The mRNA level of *MyHC I*, slow muscle fiber marker, was significantly higher in soleus (SOL) than in extensor digitalis longus (EDL), whereas the mRNA of *MyHC IIB*, fast muscle fiber marker, was significantly higher in EDL than in SOL (Fig 1*A*). Then, the expression of lnc-H19 in SOL and EDL was detected, and it was found that the expression of lnc-H19 transcripts in SOL was significantly higher than that in EDL (Fig. 1*B*). In addition, lnc-H19 expression gradually increased during C2C12 myoblast differentiation and remained at a high level at later stages (Fig. 1*C*). This suggests that lnc-H19 may play an important role in the transformation of skeletal muscle fiber types.

### Lnc-H19 promoted the conversion of fast muscle fibers to slow muscle fibers

**Figure 2:**
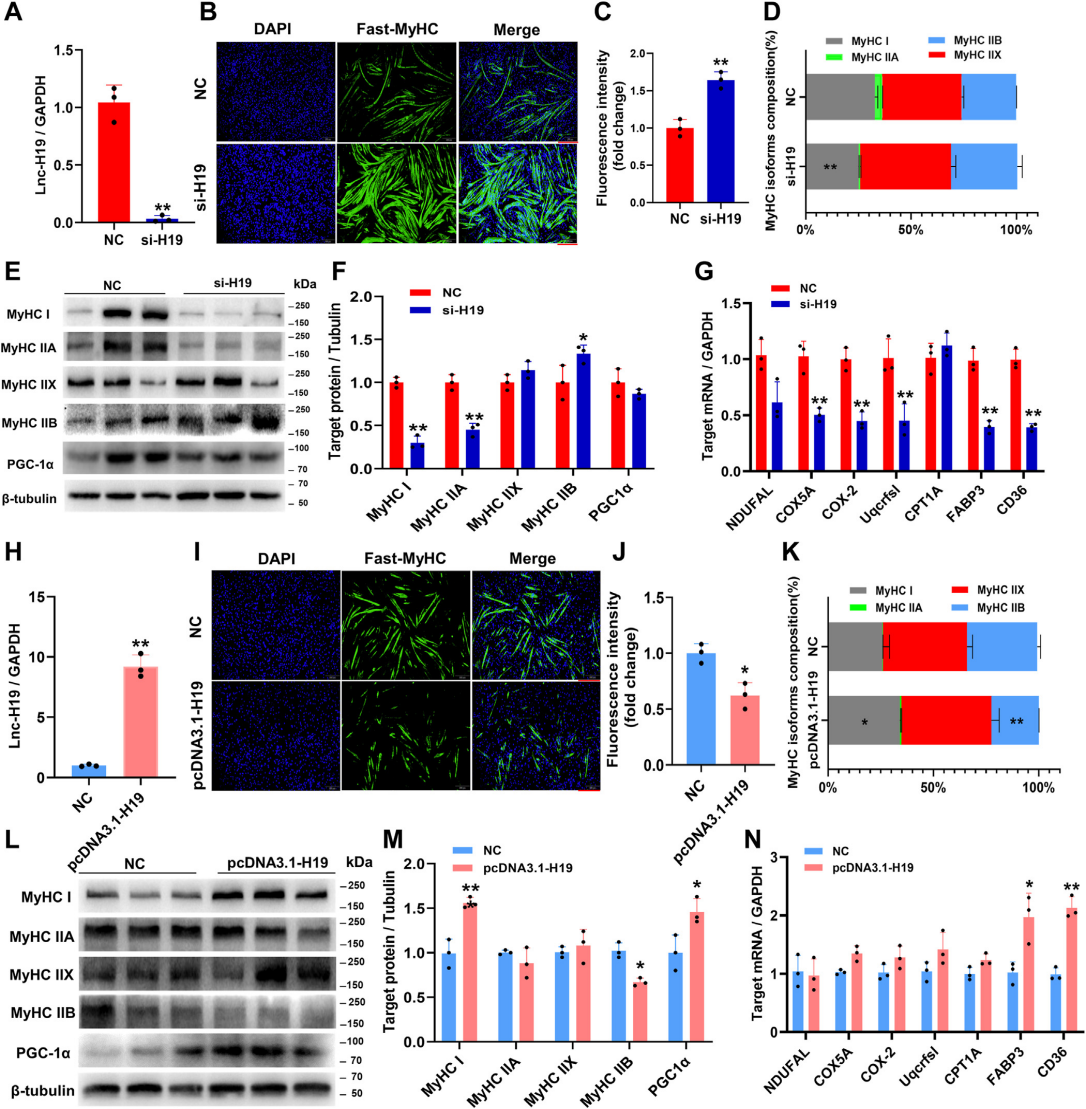
**Lnc-H19 promotes the conversion of fast muscle fibers to slow muscle fibers in C2C12 cell.***A*, the mRNA expression level of lnc-H19 was interfered (n = 3 biological replicates). *B*, immunofluorescence staining of fast-MyHC after interference of lnc-H19 (scale bar represents 200 μm). Each group of three biological repeats. *C* and *J*, *B* and *I*, statistics of immunofluorescence intensity. Five views per photo were selected and counted, and the average was set as the value of this sample statistical analysis (n = 3 biological replicates). *D* and *K*, MyHC isoform composition (%) (n = 3 biological replicates). *E* and *L*, the protein expression levels of MyHCI, MyHC IIA, MyHC IIX, and MyHC IIB (n = 3 biological replicates). *F* and *M,* quantification of MyHC I, MyHC IIA, MyHC IIX, MyHC IIB, and PGC-1α protein (n = 3 biological replicates). *G* and *N*, the expression of mitochondrial-related genes (n = 3 biological replicates). *H*, the mRNA expression level of lnc-H19 was overexpressed (n = 3 biological replicates). *I*, immunofluorescence staining of fast-MyHC after overexpression of lnc-H19 (scale bar represents 200 μm). Each group of three biological repeats. The results are expressed as mean ± SD, ∗*p* < 0.05 and ∗∗*p* < 0.01. lnc, long noncoding.

To further reveal the role of lnc-H19 in the process of muscle fiber type transition, si-H19 were transfected into C2C12 myoblasts utilizing scramble siRNAs as negative control. The results of quantitative RT–PCR (qRT–PCR) showed that the lnc-H19 transcripts were reduced by about 90% compared with the control group (Fig. 2*A*). Immunofluorescence staining showed that lnc-H19 interference significantly promoted the formation of fast-MyHC myotubes (Fig. 2, *B* and *C*). Meanwhile, qRT–PCR and Western blot results showed that downregulation of lnc-H19 significantly decreased the mRNA and protein levels of slow muscle fiber marker *MyHC I* and dramatically increased the expressions of *MyHC IIB* (Fig. 2, *D*–*F*). Moreover, lnc-H19 inhibition significantly suppressed the expression of mitochondrial-related genes, including cytochrome *c* oxidase subunit Va (*COX5A*), cyclooxygenase-2 (*COX-2*), ubiquinol–cytochrome *c* reductase, Rieske iron–sulfur polypeptide (*UQCRFSL*), fatty acid binding protein 3 (*FABP3*), and cluster of differentiation 36 (*CD36*) at the mRNA level (Fig. 2*G*). Inversely, pcDNA3.1-H19 powerfully upregulated lnc-H19 expression by approximately eightfold (Fig. 2*H*). Overexpression of lnc-H19 significantly inhibited the generation of fast-MyHC myotubes (Fig. 2, *I* and *J*) and the mRNA and protein expression levels of the fast muscle fiber gene *MyHC IIB* (Fig. 2, *K* and *M*) and promoted the expressions of slow muscle fiber gene *MyHC I* and mitochondria-related genes *FABP3* and *CD36* (Fig. 2*N*). These results suggested that lnc-H19 might promote the transition of fast muscle fibers into slow muscle fibers with altered mitochondrial function.

### Lnc-H19 knockdown *in vivo* decreased endurance exercise in mice

**Figure 3:**
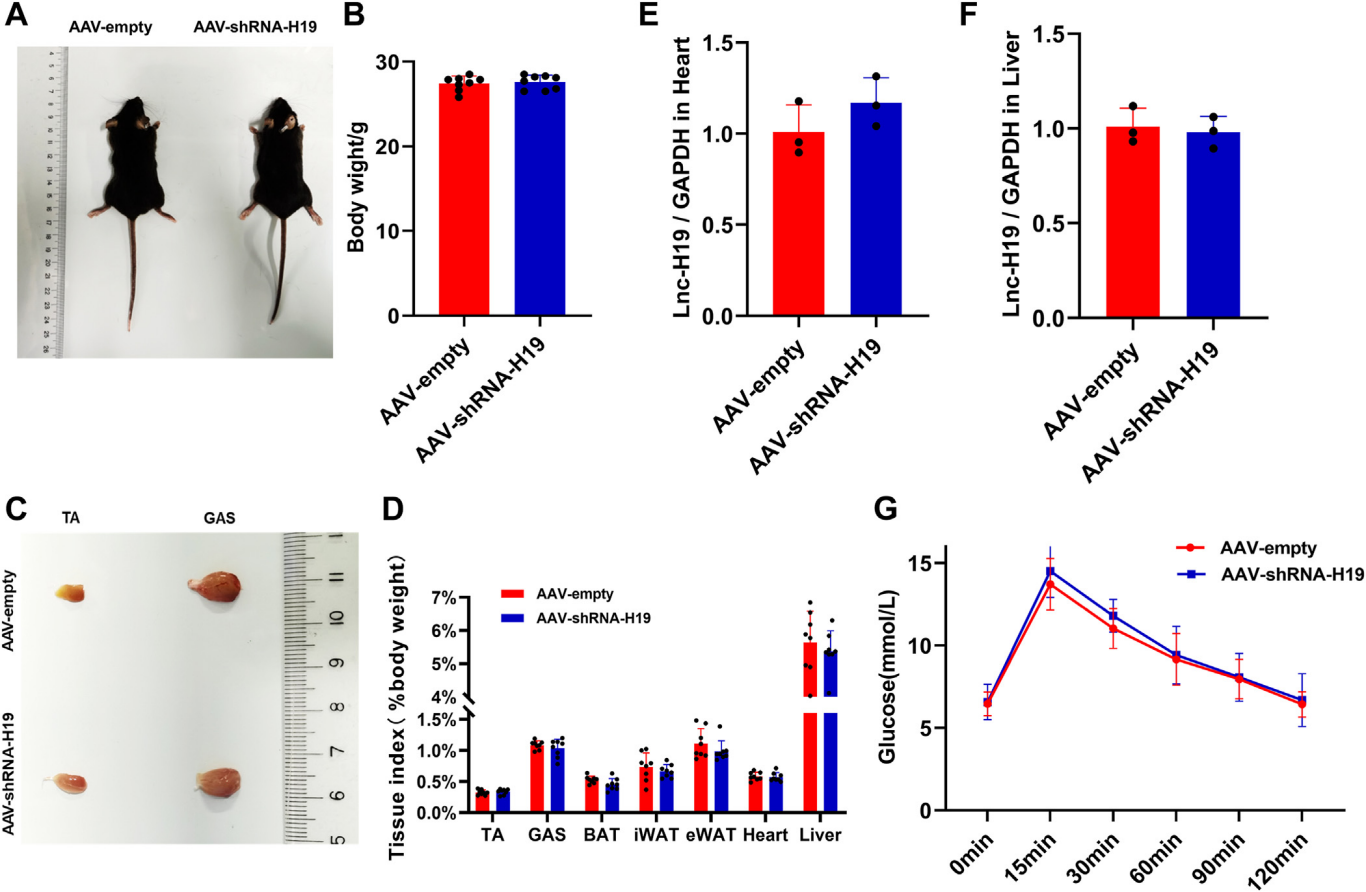
**Effect of AAV-shRNA-H19 injection on body condition and glucose tolerance test in mice**. *A*, gross view of AAV-empty and AAV-shRNA-H19 mice. *B*, body weight of AAV-empty and AAV-shRNA-H19 injected mice (n = 8 biological replicates). *C*, gross view of TA and GAS from AAV-empty and AAV-shRNA-H19 mice. *D*, si-H19 mice for each tissue weight to mice body weight ratio (%) (n = 8 biological replicates). *E*, expression of lnc-H19 in the heart in mice (n = 3 biological replicates). *F*, expression of lnc-H19 in the liver in mice (n = 3 biological replicates). *G*, glucose tolerance of AAV-empty and AAV-shRNA-H19 mice (n = 8 biological replicates). The results are expressed as mean ± SD, ∗*p* < 0.05 and ∗∗*p* < 0.01. AAV, adeno-associated virus; GAS, gastrocnemius; lnc, long noncoding; TA, tibialis anterior.

To determine the role of lnc-H19 in muscle fiber type conversion *in vivo*, 8-week-old male C57/BL6J mice were injected with AAV-shRNA-H19 with AAV-empty as control. Compared with the control counterparts, there was no significant difference neither in body weight (Fig. 3, *A* and *B*) nor in the size and mass of the tibialis anterior (TA) or gastrocnemius (GAS) of the AAV-shRNA-H19 knockdown mice (Fig. 3, *C* and *D*). In addition, the expression of lnc-H19 in heart and liver was not significantly different after injection of AAV-shRNA-H19 (Fig. 3, *E* and *F*). Furthermore, the glucose tolerance (Fig. 3*G*) and serum biochemical index (Fig. S1) were not significantly different between groups. The aforementioned results showed that the deficiency of lnc-H19 in muscle exerted no great effects on the body condition and physiological status in mice.

**Figure 4:**
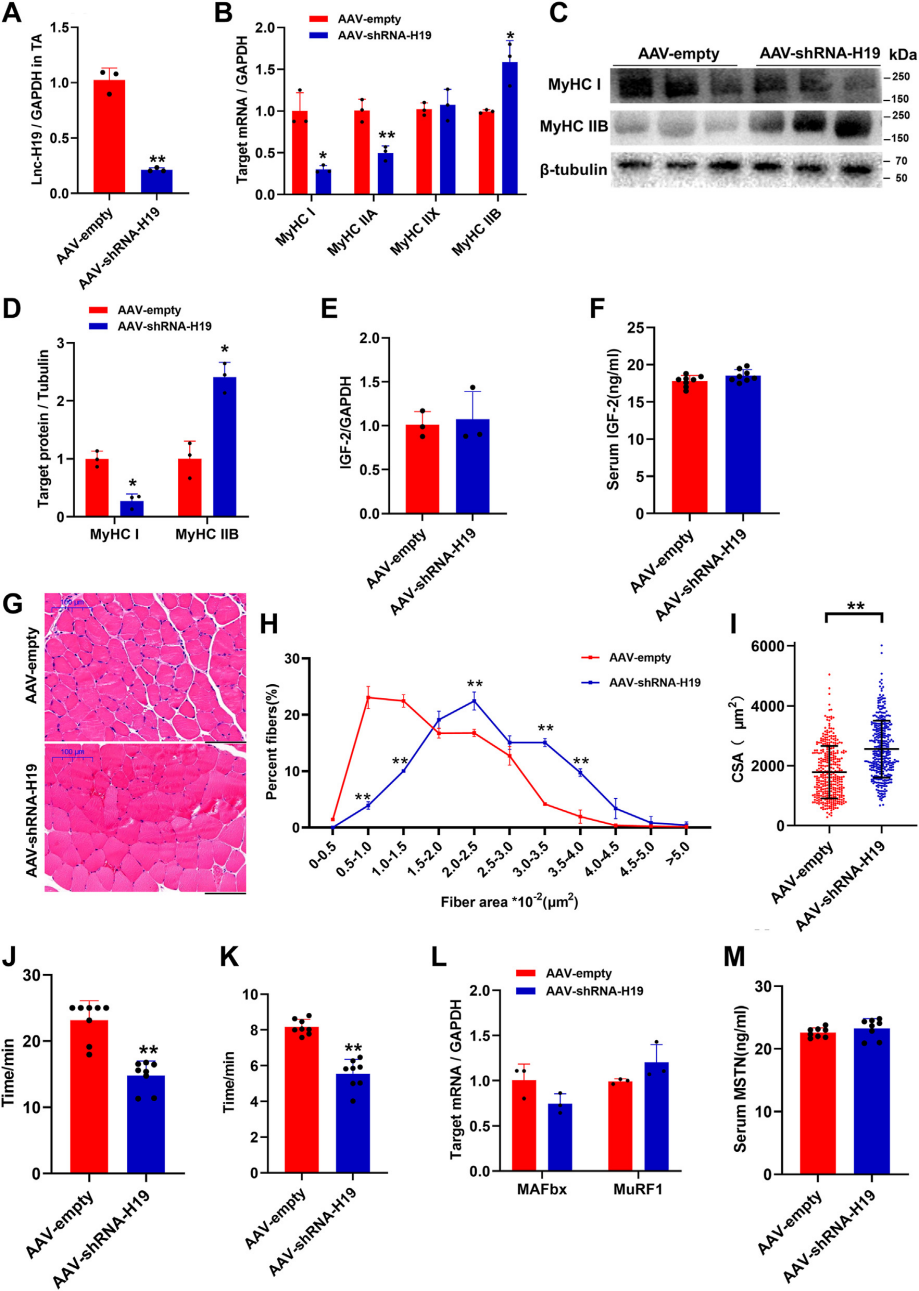
**Interference with lnc-H19 affected the muscle fiber type and exercise ability of TA.***A*, interference efficiency of lnc-H19 in TA (n = 3 biological replicates). *B*, the expression of MyHC isoforms in TA (n = 3 biological replicates). *C*, statistical results of MyHC I and MyHC IIB muscle proteins (n = 3 biological replicates). *D*, MyHC I and MyHC IIB muscle protein expression levels (n = 3 biological replicates). *E*, the expression of *IGF-2* in TA (n = 3 biological replicates). *F*, the content of IGF-2 in serum (n = 8 biological replicates). *G*, H&E staining of TA muscle in mice (n = 3 biological replicates). *H* and *I*, cross-sectional area muscle fiber of TA muscle in mice. n = 3, three fields were randomly taken from each mouse H&E sections for CAS statistics. *J*, weight-bearing swimming time in mice (n = 8 biological replicates). *K*, limb suspension time of mice (n = 8 biological replicates). *L*, the expression of *MAFbx* and *MuRF1* (n = 3 biological replicates). *M*, the content of MSTN in serum (n = 8 biological replicates). The results are expressed as mean ± SD, ∗*p* < 0.05 and ∗∗*p* < 0.01. CAS, Chemical Abstracts Service; IGF-2, insulin-like growth factor 2; lnc, long noncoding; MSTN, myostatin; TA, tibialis anterior.

After injection of AAV-shRNA-H19, lnc-H19 mRNA was significantly inhibited in TA (Fig. 4*A*) and GAS (Fig. S2*A*). In addition, the mRNA expression of slow muscle fiber gene *MyHC I* was inhibited, whereas the expression of fast muscle fiber gene *MyHC IIB* was significantly increased (Figs. 4*B* and S2*B*). Western blot results showed that MyHC I was significantly increased and MyHC IIB was decreased at protein levels (Fig. 4, *C* and *D*). These results indicated that interference of lnc-H19 could promote the transition of slow-to-fast muscle fibers. Considering that *IGF-2* (insulin-like growth factor 2) is the linked imprint gene of lnc-H19 on mouse chromosome 7, both may have synergistic effects during development (15). Therefore, the mRNA expression in TA and serum concentration of *IGF-2* were detected, and there was no significant change in the expression of IGF-2 after interference of lnc-H19 (Fig. 4, *E* and *F*). H&E staining showed that the cross-sectional area of TA in the AAV-shRNA-H19 mice was significantly larger than that in the AAV-empty group (Fig. 4, *G*–*I*). Besides, swimming time and limb hanging time in AAV-shRNA-H19 group were significantly lower than those of the control (Fig. 4, *J* and *K*). Further results showed that there was no significant difference in the mRNA levels of *MAFbx*, *MuRF*1, and serum myostatin contents (Fig. 4, *L* and *M*). In summary, we demonstrated that interference of lnc-H19 promoted fast muscle fiber formation in mice, which is not associated with amyotrophy.

### Lnc-H19 promoted the formation of slow muscle fiber through affecting the morphology and function of mitochondria

**Figure 5:**
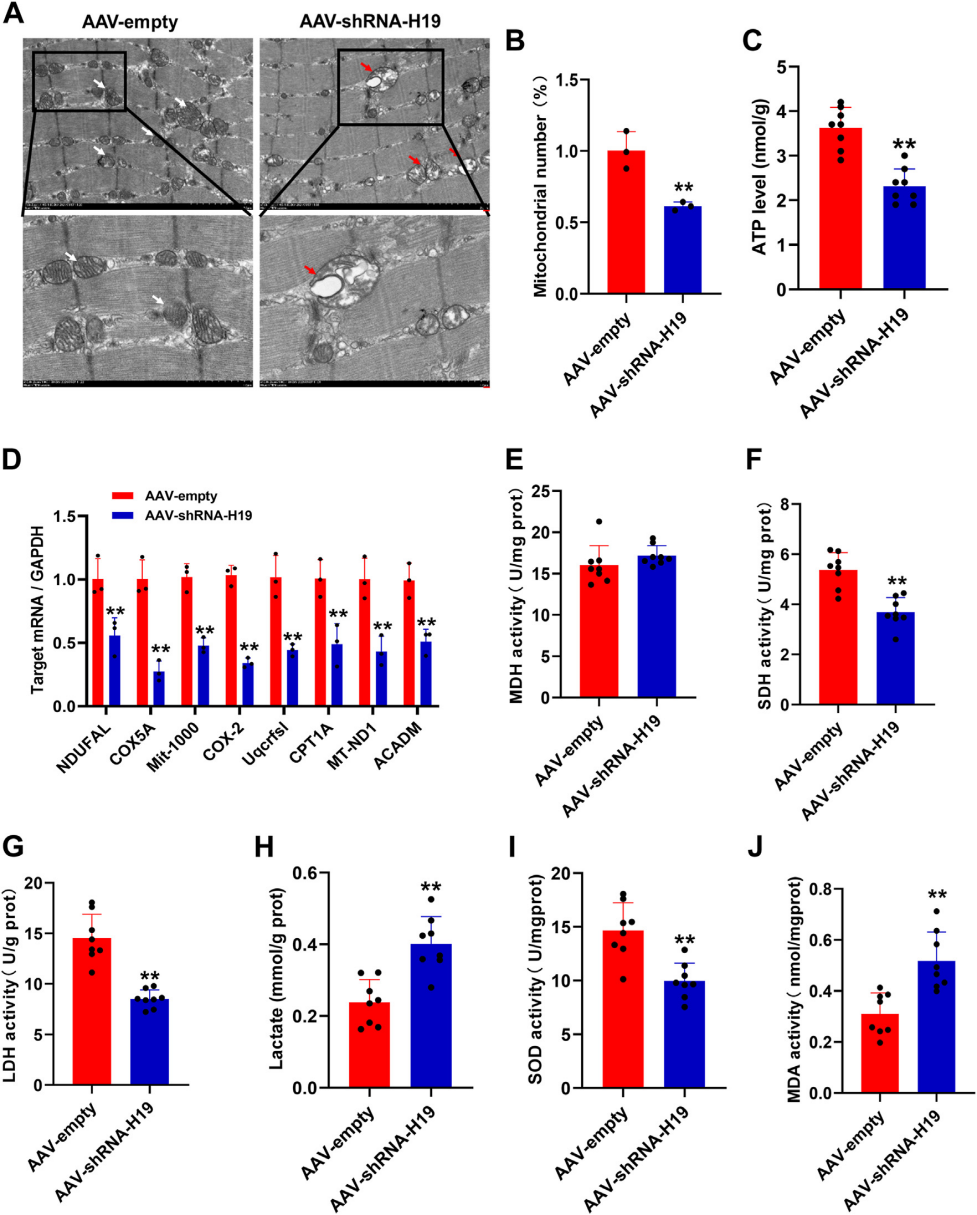
**Morphology and function of mitochondria in TA of mice**. *A*, transmission electron microscope image, *Top left* and *top right* magnifications = 7000, scale bar represents 1 μm; *lower left* and *lower right* magnifications = 15,000, scale bar represents 2 μm. *B*, mitochondrial count in three mice, each mouse selected three visual fields, and counted the number of mitochondria for significance analysis. *C*, ATP level in TA (n = 8 biological replicates). *D*, the mRNA levels of mitochondria-related genes (n = 3 biological replicates). *E*, the MDH activity in GAS (n = 8 biological replicates). *F*, the SDH activity in GAS (n = 8 biological replicates). *G*, the LDH activity in GAS (n = 8 biological replicates). *H*, the lactate content in GAS (n = 8 biological replicates). *I*, the SOD activity in GAS (n = 8 biological replicates). *J*, the MDA content in GAS (n = 8 biological replicates). The results are expressed as mean ± SD. ∗*p* < 0.05 and ∗∗*p* < 0.01. GAS, gastrocnemius; LDH, lactic dehydrogenase; MDA, malondialdehyde; MDH, malate dehydrogenase; SDH, succinate dehydrogenase; SOD, superoxide dismutase; TA, tibialis anterior.

Slow muscle fiber contains a large number of mitochondria, which are mainly metabolized by oxidation, whereas fast muscle fiber has a small number of mitochondria, which are mainly metabolized by glycolysis. In order to clarify the effect of lnc-H19 on mitochondria in muscle, transmission electron microscopy was performed on the TA muscles. After AAV-shRNA-H19 injection, morphology of mitochondria presented swelling and vacuolation (Fig. 5*A*), and the number of mitochondria in TA was significantly decreased (Fig. 5*B*), and ATP content was significantly decreased (Fig. 5*C*). Meanwhile, qRT–PCR results showed that the expression of mitochondria-related genes, including *NDUFAL*, *COX5A*, *Mit-1000*, *COX-2*, *UQCRFSL*, *CPT1A*, *MT-ND1*, and *ACADM*, was significantly decreased after lnc-H19 knockdown *in vivo* (Fig. 5*D*). Furthermore, the deletion of lnc-H19 did not greatly alter malate dehydrogenase activity (Fig. 5*E*) but significantly decreased succinic dehydrogenase and lactate dehydrogenase activities (Fig. 5, *F* and *G*). The content of lactate, a metabolite of glycolysis, was significantly increased (Fig. 5*H*). Besides, lnc-H19 knockdown significantly reduced the activity of SOD (Fig. 5*I*) and increased the content of malondialdehyde (Fig. 5*J*). These results indicated that the deficiency of lnc-H19 affected the morphology and function of mitochondria and changed the metabolic pattern of muscle.

## Discussion

The roles of lncRNAs in skeletal muscle development have been intensively reported. For example, lnc-H19 can regulate the differentiation of porcine satellite cells through miR-140-5p/SOX4 and DBN1 (16), and lncRNA Has2os promotes myoblast differentiation through the c-Jun N-terminal kinase–mitogen-activated protein kinase signaling pathway (17). In addition, lncRNAs have been reported to modulate transformation of muscle fiber type, for there is great difference of lncRNA profiles between fast and slow muscle fibers (18). For example, lnc-MyH, located within *MyHC IIA* gene loci, could inhibit the proportion of slow muscle fibers *in vitro* (19). In addition, porcine MyHC IIA/X-AS, mainly located in the intergenic region between *MyHC IIA* and *IIX*, has been reported to maintain the phenotype of fast muscle fiber types through sponging miR-130b (10). Muscle-specific knockout of lnc-mg, partially overlapping *MyHC IIX* gene in mice, resulted in weakening of muscle endurance exercise in mice (20). Using loss- and gain-of-function strategies, our work suggested that lnc-H19 is important for the maintenance of the slow muscle phenotype *in vitro*.

Because of the differences between *in vivo* and *in vitro*, we studied the effect of lnc-H19 knockdown on transformation of muscle fiber types by *in vivo* injection of AAV9 constructs. Consistent with the results in C2C12 cells, lnc-H19 knockdown in mouse skeletal muscle specifically promoted fast muscle fiber generation and decreased slow muscle fiber composition. Slow muscle fibers contain a large number of mitochondria, which contribute to durable and slow contraction, and individuals with more slow muscle fibers exhibited better performance of endurance exercise (21). Through weight-bearing swimming experiment and limb suspension experiment, we found that muscle-specific knockdown of lnc-H19 significantly reduced swimming duration and suspension time of mice, indicating that the endurance exercise of these mice was impaired. Of note, we also examined the expression of genes related to muscle atrophy and found that the damaged endurance exercise was not related with muscle atrophy.

Mitochondrial density, a classic and unique feature to distinguish fiber types, typically displays two to three times more in slow-twitch type I fibers than fast-twitch type II fibers, following with significantly elevated oxidative ATP synthesis capacity (22). Oxidative phosphorylation mainly occurs in mitochondria, and the more mitochondria there are, the stronger energy metabolism will be. Glycolysis occurs primarily in the cytoplasm, where energy is supplied by a series of enzymes that catalyze glycolysis reactions (23). It has been reported that lnc-H19 inhibits mitochondrial apoptosis of cardiomyocytes to alleviate heart defects (24), and lnc-H19 also repressed mitochondrial dysfunction and reduced reactive oxygen species production (25). In addition, H19 depletion leads to impaired insulin signaling and reduced glucose uptake (26). Interestingly, we found that lnc-H19 knockdown resulted in a significant reduction in the number of skeletal muscle mitochondria, swelling of mitochondria, and vacuolation. Meanwhile, lnc-H19 knockdown promoted lactate production, and decreased mitochondrial number and ATP production, which was consistent with the results of reduced exercise endurance in mice.

## Conclusion

Our work identified a novel function of lnc-H19 in muscle fiber type transformation both *in vivo* and *in vitro*, and muscle-specific knockdown of lnc-H19 leads to repressed endurance exercise with impaired mitochondria function.

## Experimental procedures

### Animals

Twenty experimental C57/BL6J male mice were purchased from the Medical Laboratory Animal Center of Xi'an Jiaotong University, and all experiments were approved by Ethics Committee of Animal Welfare and Health of Northwest A&F University. Three mice were killed by cervical dislocation, and SOL and EDL were collected and stored in liquid nitrogen until further analysis.

### Knockdown of lnc-H19 by AAV infection *in vivo*

Sixteen 8-week-old C57/BL6J male mice were randomly allocated into two groups, with eight mice per group. Adeno-associated viruses AAV-shRNA-H19 and AAV-empty were synthesized from HanBio. Mice were subjected to multipoint intramuscular injection with 60 μl of AAV-shRNA-H19 or AAV-empty in the TA muscle. Muscle, fat, and internal organs were collected 1 month after injection. Mice were weighted every week. Before sampling, all mice were sequentially subjected to glucose tolerance test (10 days before sampling), weight-bearing swimming (5 days earlier), and limb suspension test (2 days earlier). When sampling, the mice were killed with cervical dislocation, and then cardiac blood was taken. Three mice in each group were randomly selected for qRT–PCR and Western blot assay, and another three mice per group were selected for H&E staining and transmission electron microscopy.

### Cell culture and transfection

C2C12 myoblast cell line is cultured in a growth medium consisting of Dulbecco’s modified Eagle’s medium (Invitrogen), 10% fetal bovine serum (Gibco), and 1% penicillin–streptomycin. When the cell density reached to 80 to 90%, the cells were switched to a differentiation medium containing 2% horse serum (Solarbio), 1% penicillin–streptomycin, and Dulbecco’s modified Eagle’s medium, which was used to induce differentiation. The cells were cultured in a cell incubator at a constant temperature and humidity (37 °C and 5% CO_2_), and medium was changed every 2 days. Lipofectamine 2000 reagent (Invitrogen) was used to transfect si-RNAs (RiboBio) or pcDNA3.1 constructs into cells in accordance with the manufacturer's protocol. According to the nucleotide sequence of lnc-H19 (NR_130973.1) in the National Center for Biotechnology Information database, pcDNA3.1-H19 was synthesized by general biotechnology. Transfection was done in triple. To avoid the possible effects of lnc-H19 on myogenic differentiation, transfection was done on myotubes 3 days postinduction, and 4 days later, cells were harvested for further analysis.

### RNA extraction, complementary DNA synthesis, and qPCR analysis

Trizol (Vazyme) reagent was used to extract total RNA from C2C12 cells. The integrity of RNA was tested by agarose gel electrophoresis, the concentration of RNA was determined by Nanodrop ND-1000 (ThermoFisher) tester, and RNA with absorbance at 260 nm/absorbance at 280 nm between 1.8 and 2.0 was used to synthesize complementary DNA (cDNA) using HiScript III first Strand cDNA Synthesis Kit (Vazyme) containing DNase to remove genomic DNA, according to the manufacturer's instructions. qRT–PCR for mRNA levels was performed on a Bio-Rad CFX96 system using SYBR real-time PCR mixture (BioTeke). The primers are shown in Table 1. Each sample was repeated three times, and the data were analyzed using 2-δδCt method, with ∗ indicates *p* < 0.05 and ∗∗ indicates *p* < 0.01.

**Table 1:** Primer sequence

Gene symbol	Forward primer	Reverse primer
mus-H19	TGAGTTTCTAGGGAGGGAG	ATTCCTGAGGCAGGTAGTG
mus-MyHC I	ACTGTCAACACTAAGAGGGTCA	TTGGATGATTTGATCTTCCAGGG
mus-MyHC IIA	AAGTGACTGTGAAAACAGAAGCA	GCAGCCATTTGTAAGGGTTGAC
mus-MyHC IIX	TTGAAAAGACGAAGCAGCGAC	AGAGAGCGGGACTCCTTCTG
mus-MyHC IIB	GCGAATCGAGGCTCAGAACAA	GTAGTTCCGCCTTCGGTCTTG
mus-NDUFA1	TTATGGGGGTGTGCTTGGTC	GTTTTTCCTTGCCCCCGTTG
mus-COX5A	TGTCTGTTCCATTCGCTGCT	AACCGTCTACATGCTCGCAA
mus-COX2	AGTTGATAACCGAGTCGTTCTG	CTGTTGCTTGATTTAGTCGGC
mus-UQCRFS1	TACAGATGTCAAGGTGCCCG	TTTGGCCGCATAAGCAACAC
mus-CPT1A	GAGACAGGACACTGTGTGGGTGA	AGTGCCTTGGCTACTTGGTACGAG
mus-FABP3	TTCTGGAAGCTAGTGGACAG	TGATGGTAGTAGGCTTGGTCAT
mus-ACADM	GTCGCCCCGGAATATGAC	ACCCATACGCCAACTCTTCG
mus-CD36	TCATATTGTGCTTGCAAATCCAA	TGTAGATCGGCTTTACCAAAGATG
mus-MTND1	GTTGGTCCATACGGCATTTT	TGGGTGTGGTATTGGTAGGG
mus-GAPDH	TCACCACCATGGAGAAGGC	GCTAAGCAGTTGGTGGTGCA

The composition of MyHC isoforms was determined as a previous report (27). Briefly, 20 ng of reverse transcriptional RNA was used in a reaction volume of 8 μl. To amplify the GAPDH rRNA (internal control), only 20 pg was used. PCR products were amplified (50 °C, 2 min and 95 °C, 10 min, then 40 at 95 °C, and 15 s and 60 °C, 1 min) and analyzed on an RT–PCR cycler. Absolute copy numbers of MyHC transcripts and GAPDH cDNA were determined using calibration curves generated with cloned PCR fragment standards. Copy numbers of individual MyHC transcripts are given in relation to those of GAPDH cDNA.

### Western blot analysis

Cells were collected from 6-well plates, washed twice with PBS, and then lysed in 150 μl radioimmunoprecipitation assay buffer containing 1% protease inhibitor (Beyotime; catalog no.: P0013B). Western blotting was performed according to a previously established procedure (28). The primary antibodies targeted the following proteins: MyHC I (1:1000 dilution; DSHB), MyHC IIA (1:1000 dilution; DSHB), MyHC IIX (1:1000 dilution; DSHB), MyHC IIB (1:1000 dilution; DSHB), Fast-MyHC (1:1000 dilution; Sigma), Slow-MyHC (1:1000 dilution; Sigma), and β-tubulin (1:200 dilution; Santa Cruz Biotechnology). The secondary antibody is antimouse horseradish peroxidase (1:5000 dilution; Santa Cruz Biotechnology).

### Immunofluorescence staining

The cells were washed with PBS twice and fixed with 4% paraformaldehyde (Biosharp) solution for 10 to 15 min at room temperature and then washed with PBS for three times. Subsequently, cells were incubated with 100 nM glycine for 10 min, washed with PBS three times, and then blocked with blocking buffer (5% goat serum, 2% bovine serum albumin, 0.2% Triton X-100, and 0.1% sodium azide) for 30 min at room temperature. Cells were immunized with primary antibody (fast-MyHC, 1:1000 dilution, catalog no.: M4276; Sigma) at 4 °C overnight, and fluorescence-labeled secondary antibody (goat antimouse IgG/FITC; Bioss) at room temperature for 1 h. 4′,6-Diamidino-2-phenylindole (1:1000 dilution, catalog no.: D1306; Thermo Scientific) was then used for nuclear staining. Finally, photographs were taken using a fluorescent inverted microscope (Olympus). Five views per photo were selected and counted, and the average was set as the value of this sample for further statistical analysis.

### H&E staining

H&E staining of TA samples was performed as previously described (29).

### Glucose tolerance test

The mice were fasted for 12 h and intraperitoneally injected with 100 mg/ml glucose (1 g/kg body weight) for glucose tolerance test. Blood glucose was measured with a glucose meter at 0, 15, 30, 60, 90, and 120 min after injection.

### Blood biochemical index

The blood samples were stored at room temperature for 2 h and centrifuged at 3000 rpm at 4 °C for 15 min. Supernatant of 100 μl was taken, and blood biochemical indexes were detected.

### Weight-bearing swimming experiment

The weight-bearing swimming experiment was performed as previously described, with minor modifications (30). Briefly, mice were loaded with a lead block equivalent to 5% of their own weight at room temperature (25.0 ± 0.5 °C) and then placed into a swimming pool with a minimum depth of 30 cm. The timing started the moment the mice were put in pool and stopped when the mice emerged under the water longer than 5 s. After the experiment, the mice were dried with a hair dryer and put back in the cage for recovery.

### Limb suspension test

The experimental mice were suspended upside down in the middle of the wire mesh, which was at least 30 cm above the ground. The exhaustion hanging time was set as the mouse falled off the wire. The maximum suspension time is 25 min.

### ELISA

The blood samples were stored at room temperature for 2 h and centrifuged at 3000 rpm at 4 °C for 15 min to collect. IGF-2 (catalog no.: CT66393) and myostatin (catalog no.: H658-1-1; Nanjing) contents were determined by ELISA according to the manufacturer's protocol.

### Transmission electron microscopy

The TA muscles were collected and immediately fixed at 4 °C in 0.1 M sodium carbonate buffer (Na (CH_3_)_2_AsO_2_•3H_2_O) with 2.5% glutaraldehyde and 4% paraformaldehyde at pH 7.2. Transmission electron microscopy sample preparation steps included osmium tetroxide fixation, ethanol dehydration, and Eponate58 embedding. Ultrathin sections (∼70 nm thick) were observed under transmission electron microscope after ultrasection and staining.

### Determination of enzyme activity and substrate concentration

The GAS of mice was collected and ground using a high-throughput tissue grinder. The following kits were purchased from Nanjing Jiancheng Bioengineering Institute. According to the manufacturer's protocol, malate dehydrogenase (catalog no.: A021-2-1), succinic dehydrogenase (catalog no.: A022-1-1), lactate dehydrogenase (catalog no.: A020-1-2), SOD (catalog no.: A001-4-1), lactate (catalog no.: A019-2-1), and malondialdehyde (catalog no.: A003-1-2) contents were detected.

### Data analysis

Data were represented as mean ± standard error. GraphPad Prism 8.0 software (GraphPad Software, Inc) was used to analyze data. Statistical significance was calculated by *t* test analysis. *p* < 0.05 was considered to be statistically significant.

## Data availability

The data used to support the findings of this study are available from the corresponding author upon request.

## Supporting information

This article contains supporting information (Figs. S1 and S2).

## Conflict of interest

The authors declare that they have no conflicts of interest with the contents of this article.
