# Supplementary material for: The long noncoding RNA lnc-H19 is important for endurance exercise by maintaining slow muscle fiber types
Source: J Biol Chem. 2023 Sep 22;299(11):105281. doi: 10.1016/j.jbc.2023.105281 (PMC10598739; doi:10.1016/j.jbc.2023.105281)
Supplement: Supporting information [file mmc1.docx]

**Supplementary material:**

**
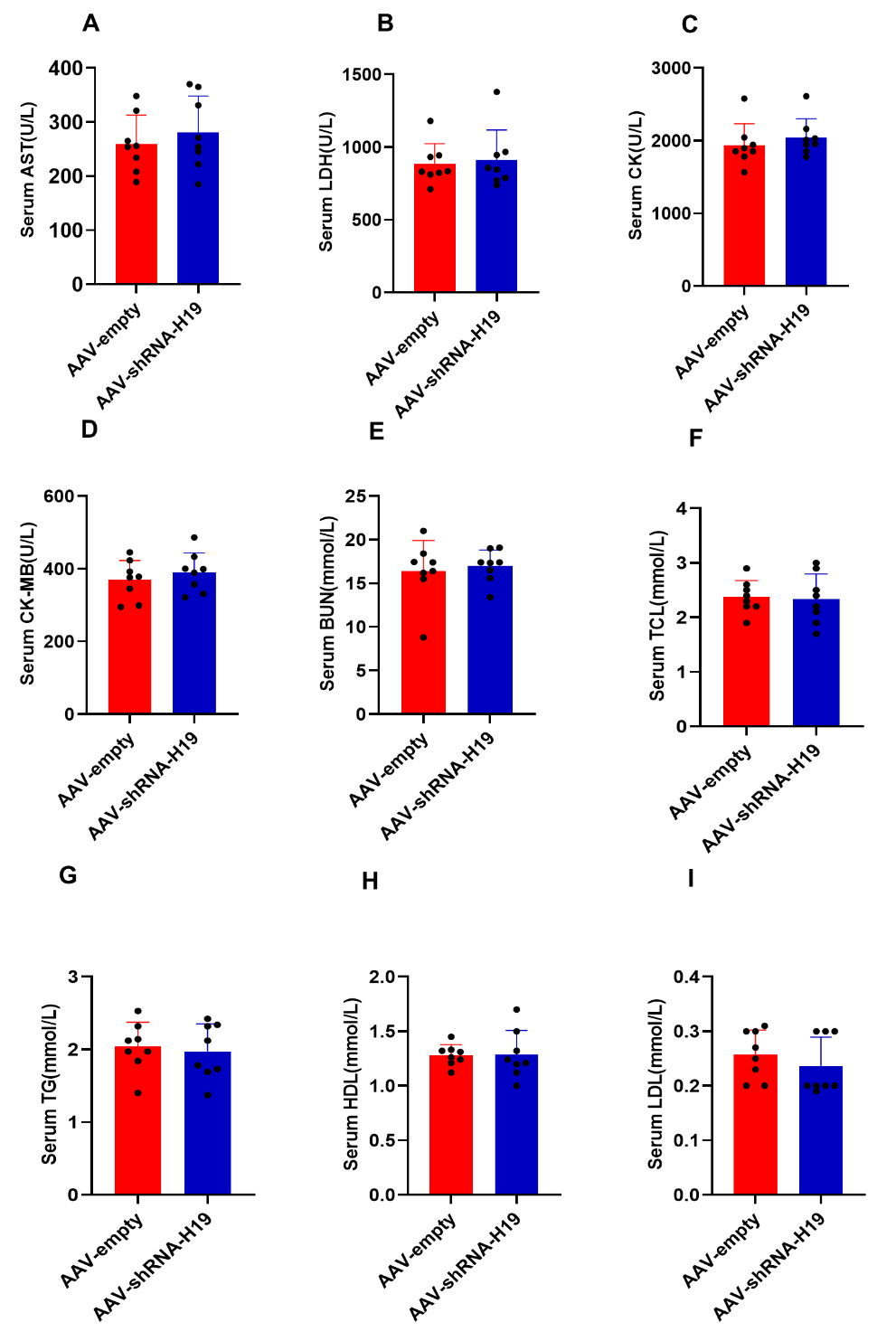
**

**Figure S1: serum biochemical index of mice A:** The concentration of Aspartate aminotransferase (AST). **B:** The concentration of lactate dehydrogenase (LDH). **C:** The concentration of creatine kinase (CK). **D:** The concentration of creatine kinase isoenzyme (CK-MB). **E:** The concentration of blood urea nitrogen (BUN). **F:** The concentration of total cholesterol (TCL). **G:** The concentration of triglyceride (TG). **H:** The concentration of high-density lipoprotein (HDL). **I:** The concentration of low-density lipoprotein (LDL). The results are expressed as mean ± SD, n=8.

**
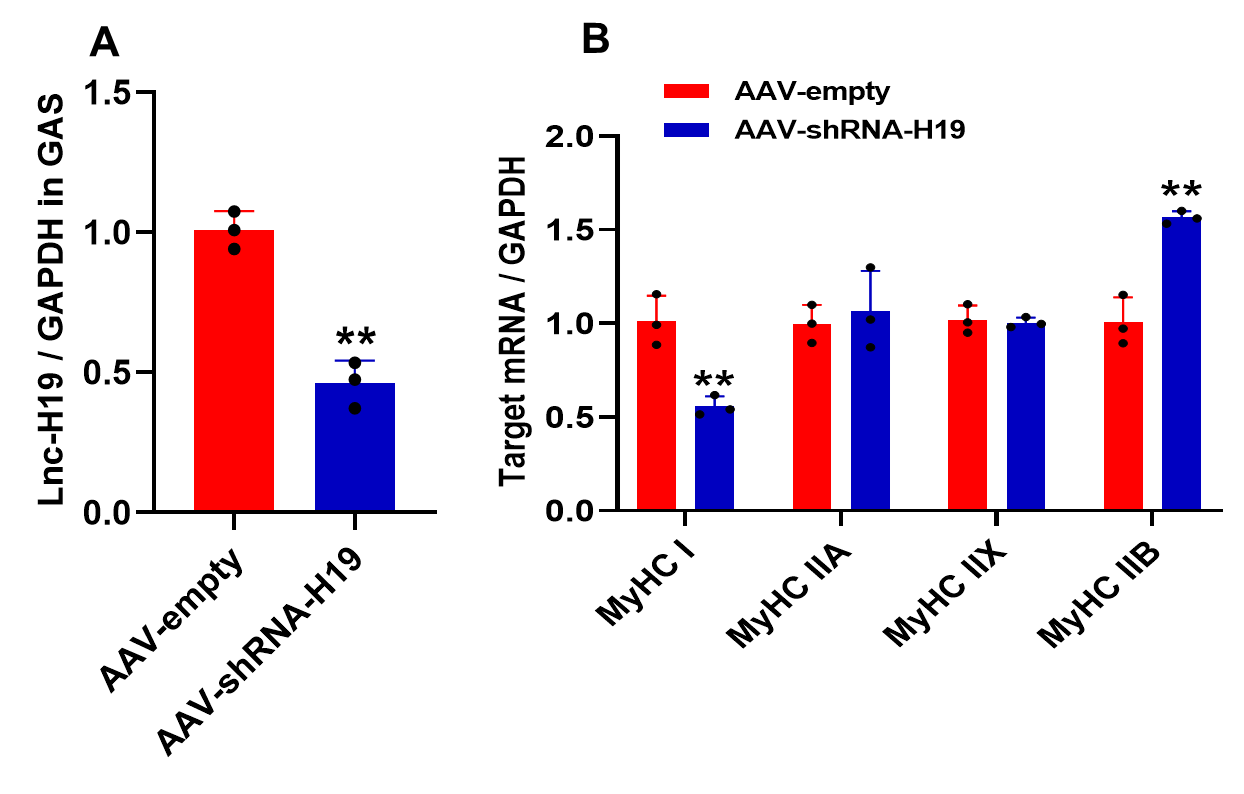
**

**Figure S2: Interference with lnc-H19 affected the muscle fiber type of GAS A:** Interference efficiency of lnc-H19 in injected GAS of mice (n = 3 biologic replicates). **B**: Expression of MyHC isoforms in GAS (n = 3 biologic replicates). The results are expressed as mean ± SD. **P* < 0.05, ***P* < 0.01.
